# Adequacy of venous thromboembolism risk stratification and prophylaxis in a tertiary university hospital

**DOI:** 10.1590/1677-5449.202300071

**Published:** 2023-07-17

**Authors:** Edvaldo Luiz Ramalli, Marcelo Bellini Dalio, Maurício Serra Ribeiro, Edwaldo Edner Joviliano

**Affiliations:** 1 Universidade de São Paulo – USP, Faculdade de Medicina de Ribeirão Preto, Departamento de Cirurgia e Anatomia, Hospital das Clínicas, Divisão de Cirurgia Vascular e Endovascular, Ribeirão Preto, SP, Brasil.

**Keywords:** venous thromboembolism, deep vein thrombosis, pulmonary embolism, risk factors, disease prevention

## Abstract

**Background:**

Venous thromboembolism is the third most common cardiovascular disease and the main cause of preventable death in hospitalized patients. Prophylaxis is still underused, despite well-established guidelines in the literature. Studies show a worldwide prophylaxis adequacy rate close to 50%.

**Objectives:**

To assess the adequacy of risk stratification and prophylactic measures for venous thromboembolism in a tertiary university hospital.

**Methods:**

A cross-sectional observational study was carried out, collecting data from medical records. Adult patients hospitalized by different specialties were enrolled and divided into surgical and clinical groups. The risk stratification of venous thromboembolism performed by the attending physicians was compared with stratification based on recent guidelines performed by the research physicians. Prophylaxis measures prescribed by the attending physicians were compared with guideline recommendations, thus obtaining the prophylaxis adequacy rate.

**Results:**

400 patients were analyzed, 169 (42.3%) surgical and 231 (57.7%) clinical. The overall stratification adequacy rate was 50.8%. Adequacy rates were 39.1% and 59.3% in the surgical and clinical groups respectively (P < 0.0001). The overall prophylaxis adequacy rate was 71.5%, with 78.1% in the surgical group and 66.7% in the clinical group (P=0.0137).

**Conclusions:**

Risk stratification adequacy is low, demonstrating a low awareness among prescribing physicians of the need for adequate stratification for prescription of prophylaxis. However, the prophylaxis prescription adequacy rates are higher than those in global data.

## INTRODUCTION

Venous thromboembolism (VTE) encompasses formation of thrombi in veins of the deep system, i.e., deep venous thrombosis (DVT), and migration of emboli to the pulmonary circulation, i.e. pulmonary thromboembolism.^1^ It is estimated that the annual incidence of VTE in Europe and the United States is 1-3 cases per 1,000 inhabitants/year.^2^ The condition is responsible for 300,000 to 600,000 admissions per year^3^ and is the third most common cardiovascular disease.^2^ There are countless risk factors for VTE, including age, immobilization, prior VTE, obesity, varicose veins, infection, cancer, chemotherapy, heart failure, pregnancy and puerperium, contraceptives, and surgical procedures, among others.

The pathophysiology of VTE is not entirely understood. However, the three pillars proposed by Rudolph Virchow in 1856 (endothelial injury, venous stasis, and hypercoagulability) are still accepted today. Endothelial injury and venous stasis are easily understood. Hypercoagulable states appear to be the key to understanding this pathophysiology. It may be possible to understand why some people suffer VTE and others do not, despite being exposed to the same risk factors, through biochemical mechanisms of cell signaling or even regulation of gene expression.^2^ ^,^ ^3^

In response to the high incidence of VTE in hospital patients and its major social and financial impacts, several methods have been developed for identifying patients with elevated risk. Predictive scores such as the Padua^4^ and Caprini et al.^3^ scores have been adopted for assessing VTE risk in patients admitted for clinical and surgical treatment respectively. Strategies have been adopted for VTE prevention based on these scores.^5^ ^,^ ^6^ Pharmaceutical and mechanical prophylaxis methods are well-established in international consensuses, both for clinical patients and surgical patients,^2^ and based on risk stratification models.^3^ ^,^ ^7^ ^,^ ^8^ However, many Brazilian^9^ ^-^ ^19^ and international publications^20^ ^-^ ^22^ show that in the real world around 50% of patients at risk of VTE do not receive prophylaxis when indicated, or receive it in an inappropriate manner. The ENDORSE cross-sectional study assessed the prevalence of inpatients at risk of VTE and the proportion of them who received the correct prophylaxis. It concluded that approximately half of these patients receive the prophylaxis recommended by the guidelines. In other words, correct VTE prophylaxis is underused worldwide.^23^

The objective of this study is to measure the true VTE prophylaxis situation in a Brazilian tertiary University hospital, evaluating the adequacy of risk stratification and prescription of prophylaxis measures.

## METHODS

### Study design and setting

A cross-sectional observational study was conducted at the Hospital das Clínicas, Faculdade de Medicina de Ribeirão Preto, in São Paulo, Brazil, from March 1, 2020, to June 30, 2020. Hospital data were used after local Ethics Committee approval (process number 15695/2011).

### Participants

Adult patients over the age of 18 years were enrolled after admission by the internal medicine, clinical surgery, gynecology and obstetrics, orthopedics, and traumatology and intensive care specialties. Patients were excluded if they had already been enrolled for a prior admission, were obese (body mass index exceeding 35 kg/m^2^), were expectant mothers, had contraindications against anticoagulants, or had indications for a vena cava filter.

### Data collected

Data were collected from patients’ electronic medical records and their respective electronic prescription records, with no prior warning to the treating medical team and without contact with patients.

### Groups

Patients were divided into two groups: clinical and surgical, based on the type of admission. Admissions were defined as clinical if the patient had not undergone any type of surgical procedure up to the time of data collection. Admissions were defined as surgical if the patient had undergone some type of procedure prior to being assessed, irrespective of the medical specialty responsible for treatment.

### VTE risk stratification

The Caprini score^3^ was used to assess VTE risk and indications for prophylaxis. The risk stratification calculated by the attending physicians at the time of hospital admission was compared to risk stratification calculated afresh by the researching physicians, based on data from the electronic medical record. The original stratifications were then classified as adequate or inadequate. These classifications were used to calculate the risk stratification adequacy rate.

### Prophylactic measures prescribed

Prescription of VTE prophylaxis measures was also evaluated, using daily medical prescription records. The following were defined as mechanical prophylaxis: early mobilization, active or passive movement of the lower limbs, elastic graduated compression stockings, and/or intermittent pneumatic compression devices. Along the same lines, pharmaceutical prophylaxis was defined as prescriptions of unfractionated heparin, low molecular weight heparin, or fondaparinux, as recommended by the Brazilian Society of Angiology and Vascular Surgery (SBACV)^5^ guidelines and the American College of Chest Physicians (ACCP) guidelines.^2^ Patients rated as low risk do not need routine pharmaceutical prophylaxis, but mechanical prophylaxis is recommended, when available. For moderate risk patients, the guidelines suggest unfractionated heparin (5000 UI every 12 hours) or low molecular weight heparin (enoxaparin 20 mg/day). Patients considered high and very high risk should be given unfractionated heparin (5000 UI every 8h) or low molecular weight heparin (enoxaparin 40 mg/day). There is no intermediate stratification for clinical patients, who are always classified as either low or high risk, according to the ACCP recommendations.

The prophylaxis measures prescribed were compared with the recommendations from the guidelines mentioned above, based on the risk stratification calculated by the researching physicians. Prophylactic measures were classified as adequate or inadequate and VTE prophylaxis adequacy rates were then calculated.

### Study size

The sample size was defined as 400 patients on the basis of a previous study.^21^ Data were collected at random, as patients were admitted during the data collection period. The representativeness of each specialty in the total sample was proportional to the number of beds allocated to each specialty in relation to the total number of beds in the hospital.

### Statistical analysis

Continuous data were expressed as mean ± standard deviation and compared using the unpaired *t* test. Categorical data were presented as absolute counts (n) and percentages (%) and compared using Fischer’s exact test. P values less than 0.05 were considered significant.

## RESULTS

### Demographics

The electronic medical records of 400 patients were analyzed, 169 (42.3%) surgical and 231 (57.7%) clinical patients. Their demographic data are shown in Table 1. The average age was 56 years, with a slight predominance of males (56.3%). Mean age was greater in the surgical group (60 years) than in the clinical group (53 years). There were no significant differences between the groups in relation to sex or skin color. The patients in the surgical group were predominantly admitted to the clinical surgery (75%) and orthopedics and traumatology (16.6%) specialties. The patients in the clinical group were mostly admitted to the internal medicine (68.4%), gynecology and obstetrics (4.8%), and intensive care (12.1%) specialties.

**Table 1:** Demographic data on all patients analyzed and by surgical and clinical groups.

		Overall (n=400)	Surgical (n=169)	Clinical (n=231)	p
Age (years), mean ± SD		56 ± 16.04	60 ± 15.51	53 ± 15.84	<0.0001∮
Male, n (%)		225 (56.3%)	93 (55%)	132 (57.1%)	0.6844*
Skin color, n (%)	White	313 (78.3%)	138 (81.7%)	175 (75.8%)	0.1778^*^
	Brown	73 (18.3%)	24 (14.2%)	39 (16.9%)	0.4905^*^
	Black	13 (3.3%)	7 (4.1%)	16 (6.9%)	0.2815^*^
	Yellow	1 (0.3%)	0 (0%)	1 (0.4%)	1^*^
Specialty, n (%)	IM	162 (40.5%)	4 (2.4%)	158 (68.4%)	<0.0001^*^
	CS	159 (39.8%)	127 (75.1%)	32 (13.9%)	<0.0001^*^
	OT	30 (7.5%)	28 (16.6%)	2 (0.9%)	<0.0001^*^
	GO	12 (3.0%)	1 (0.6%)	11 (4.8%)	0.0165^*^
	ICU	37 (9.3%)	9 (5.3%)	28 (12.1%)	0.0229^*^

SD - standard deviation; IM - internal medicine; CS - clinical surgery; OT - orthopedics and traumatology; GO - gynecology and obstetrics; ICU - intensive care unit;

∮- unpaired *t* test;

* - Fisher’s exact test.

### VTE risk stratification

Table 2 lists the results of VTE risk stratification calculated by the attending physicians and by the researching physicians. The attending physicians classified a majority of the patients (56.3%) as at low risk, in both the surgical group (55%) and the clinical group (57.1%). The researching physicians considered the majority of the whole sample (72%) to be high risk and also in both groups, surgical (76.3%) and clinical (68.8%) (p < 0.0001). The overall stratification adequacy rate was 50.8%. Adequacy rates in the surgical and clinical groups were 39.1 and 59.3% respectively (p < 0.0001).

**Table 2:** Venous thromboembolism risk stratification conducted by attending physicians and by researching physicians for all patients and by surgical and clinical groups, with corresponding adequacy rates.

	VTE risk	Attending physicians n (%)	Researching physicians n (%)	p*	Adequacy rates (%)
Overall (n=400)	Low	225 (56.3%)	89 (22.3%)	<0.0001	50.8%
	Moderate	31 (7.8%)	23 (5.8%)	0.3239	
	High	144 (36%)	288 (72%)	<0.0001	
Surgical (n=169)	Low	93 (55%)	17 (10.1%)	<0.0001	39.1%
	Moderate	31 (18.3%)	23 (13.6%)	0.2987	
	High	45 (26.6%)	129 (76.3%)	<0.0001	
Clinical (n=231)	Low	132 (57.1%)	72 (31.2%)	<0.0001	59.3%
	Moderate	-	-	-	
	High	99 (42.9%)	159 (68.8%)	<0.0001	

VTE - venous thromboembolism;

* - Fisher’s exact test.

### Prophylactic measures prescribed

Table 3 lists the VTE prophylaxis measures prescribed by the attending physicians. Enoxaparin was the most frequent option (45%), followed by unfractionated heparin (19%) and mechanical prophylaxis (15%). One patient was prescribed rivaroxaban (0.3%), which conflicts with the recommendations of the guidelines. All of the patients given pharmaceutical prophylaxis also received mechanical prophylaxis. No form of prophylaxis whatsoever was prescribed to 20.5% of the patients. Enoxaparin was also the most used method in the surgical group (51.5%), followed by unfractionated heparin (20.1%), mechanical prophylaxis (16.6%), and no prophylaxis (11.8%). In the clinical group, enoxaparin accounted for 40.3%, unfractionated heparin for 18.2%, mechanical prophylaxis for 13.9%, fondaparinux for 0.4%, and rivaroxaban (off label) for 0.4%. No form of prophylaxis whatsoever was prescribed to 26.8% of the patients.

**Table 3:** Venous thromboembolism prophylaxis measures prescribed by the attending physicians for all patients and by surgical and clinical groups, with corresponding adequacy rates.

	VTE prophylaxis measures	Attending physicians n (%)	Adequacy rates (%)
Overall (n=400)	None	82 (20.5%)	71.5%
	Mechanical	60 (15%)	
	Enoxaparin	180 (45%)	
	UFH	76 (19%)	
	Rivaroxaban	1 (0.3%)	
	Fondaparinux	1 (0.3%)	
			
Surgical (n=169)	None	20 (11.8%)	78.1%
	Mechanical	28 (16.6%)	
	Enoxaparin	87 (51.5%)	
	UFH	34 (20.1%)	
	Rivaroxaban	0 (0%)	
	Fondaparinux	0 (0%)	
			
Clinical (n=231)	None	62 (26.8%)	66.7%
	Mechanical	32 (13.9%)	
	Enoxaparin	93 (40.3%)	
	UFH	42 (18.2%)	
	Rivaroxaban	1 (0.4%)	
	Fondaparinux	1 (0.4%)	

VTE - venous thromboembolism; UFH - unfractionated heparin.

Adequacy of prophylactic measures was analyzed individually. The overall rate of adequacy was 71.5%, with 78.1% in the surgical group and 66.7% in the clinical group (p = 0.0137). Analysis of the reasons for inadequacy identified four different scenarios: 1) patients who did not receive any type of prophylaxis; 2) patients who should have received pharmaceutical prophylaxis but did not; 3) patients who were given pharmaceutical prophylaxis, but did not fulfill the criteria for it; 4) patients who were given medications or doses different from those recommended. In the overall data, failure to prescribe prophylaxis was the most common error, accounting for 71% of cases of inadequacy (81/114), followed by non-prescription of pharmaceutical prophylaxis when indicated, in 28% of cases of inadequacy (32/114). There were no cases of prescription of pharmaceutical prophylaxis to patients for whom it was not indicated and just 1% of cases of inadequacy were medication or dosage errors (1/114).

The most common error in the surgical group was patients who did not receive any type of prophylaxis, accounting for 54% of cases of inadequacy (20/37). Patients who were not given pharmacological prophylaxis when indicated accounted for 46% of cases of inadequacy (17/37). In the clinical group, patients who did not receive any type of prophylaxis accounted for 79% of cases of inadequacy (61/77) and patients who were not given pharmacological prophylaxis when indicated accounted for 19% of cases of inadequacy (15/77), while patients given incorrect medication accounted for 1% of cases of inadequacy (1/77). Table 4 summarizes these results.

**Table 4:** Analysis of the adequacy of thromboembolism venous risk stratification and prophylaxis prescribed by attending physicians, according to guideline recommendations, for all patients and in the surgical and clinical groups, with corresponding adequacy rates.

		Overall (n=400)	Surgical (n=169)	Clinical (n=231)	*P* *
VTE risk stratification adequacy rates (%)		50.8%	39.1%	59.3%	<0.0001
VTE prophylaxis adequacy rates (%)		71.5%	78.1%	66.7%	0.0137
Did not receive adequate prophylaxis, n (%)		114 (28.5%)	37 (21.9%)	77 (33.3%)	0.0137
Type of inadequacy:					
	Did not receive any prophylaxis, n (%)	81 (71%)	20 (54%)	61 (79%)	0.0080
	Did not receive pharmaceutical prophylaxis when indicated, n (%)	32 (28%)	17 (46%)	15 (19%)	0.0068
	Received pharmaceutical prophylaxis when not indicated, n (%)	0 (0%)	0 (0%)	0 (0%)	1
	Received inadequate medication/dose, n (%)	1 (1%)	0 (0%)	1 (1%)	1

VTE- venous thromboembolism;

* - Fisher’s exact test.

## DISCUSSION

The benefits of adequate VTE prophylaxis in patients admitted to hospital are proven and prophylaxis is more opportune than VTE treatment. Ideally, all patients admitted to hospital should be stratified for risk of developing thromboembolic phenomena and receive the appropriate prophylaxis to prevent them. The role played by attending physicians in this process is fundamental.^1^ ^,^ ^24^ The present study demonstrated discrepancies between the recommendations for thromboprophylaxis based on scientific evidence and current clinical practice. This result is in agreement with worldwide published data.^23^

In the present study, the attending physicians’ VTE risk stratification adequacy rate was 50.8%. As such, approximately half of the patients admitted were not stratified correctly, even though the electronic prescription system has a tool specifically for this calculation. In the surgical group, adequacy rates were even lower (39.1%). Low risk stratification adequacy rates show that the attending physicians have poor compliance with filling out the institution’s VTE prevention documentation. Despite the availability of a digital tool that simplifies risk stratification in an intuitive and practical manner, a large proportion of the attending physicians do not correctly input the information into the application and probably simply skip steps, underestimating the VTE risk stratification of their patients.

Comparing the adequacy rate the VTE prophylaxis measures prescribed by the attending physicians with those recommended in the SBACV and ACCP guidelines, based on the risk stratification calculated by the researching physicians, it was observed that was 71.5%. This rate is significantly higher than rates reported in similar studies, such as the ENDORSE study, which found a global prophylaxis adequacy rate of 49.8%. Comparing the data from the present study with the Brazilian data from the ENDORSE study, it is notable that we observed prophylaxis adequacy rates of 78.1% and 66.7% in the surgical group and clinical group respectively, whereas the ENDORSE study reported 46 and 59%, respectively. We therefore observe an important increase in adequacy rates over time in these data from Brazilian health care services.^23^ In another, more recent Brazilian study conducted at another Brazilian University Hospital with similar characteristics to ours, Curtarelli et al. found evidence of overall VTE prophylaxis adequacy rates of 42.1%, with 37.5% in the surgical group and 52.9% in the clinical group.^25^ A study by Bastos et al.^26^ demonstrated that pharmaceutical DVT prophylaxis is employed in just 50% of patients with indications for its use, even in university hospitals.

Analysis of the reasons for prophylaxis prescription inadequacy identified the four scenarios described above. The first situation, in which the patient did not receive any type of VTE prophylaxis measure, was the most common, accounting for 71% of cases of inadequacy, 54% in the surgical group and 79% in the clinical group. Patients who did not receive pharmacological prophylaxis when indicated accounted for 28% of cases of inadequacy, 46% in the surgical group and 19% in the clinical group. Patients given incorrect medication or dosage accounted for 1% and no patients were given pharmaceutical prophylaxis without indications for it. These data on the reasons for prophylaxis prescription inadequacy are close to those observed by Curtarelli et al. in a similar analysis.^25^

An intriguing finding in the present study is the disconnect between the risk stratification adequacy rate and the prophylaxis prescription adequacy rates. Whereas the stratification adequacy rates were around 50%, the prophylaxis adequacy rates exceeded 70%. In the surgical group, this disconnect was even greater, with stratification adequacy close to 40%, whereas prophylaxis adequacy was approaching 80%. This finding reveals the prescribing physicians’ low rate of compliance with completion of the institutional documentation on VTE prevention. As such, the auxiliary methods for risk stratification calculation were being underutilized. Patient history taking and clinical examination support a professional with good medical training in VTE prophylaxis decision making. The digital resources available at the hospital support correct documentation and individual risk assessment of each patient. This can avoid the attending physicians basing their prophylaxis prescriptions on empiricism and personal perception of risk, thereby preventing occurrence of undesirable outcomes. On the other hand, the present study data reveal higher prophylaxis prescription adequacy rates than previous studies, demonstrating that clinical VTE protection practice at the institution has progressed favorably.

Considering the estimate that up to 75% of patients who suffer VTE are in hospital and that almost half of them have three or more risk factors, it is believed that many prevalent and easily identified risk factors tend not to be routinely tracked.^27^ Because they do not see these risk factors as causes of VTE, hospital services fail to provide prophylaxis. This was shown by the results of the ENDORSE study, which concluded that the majority of patients in hospital in Brazil and worldwide are at risk of developing VTE and many do not receive the recommended prophylaxis.^23^ Some authors have suggested hypotheses to explain the incorrect or absent utilization of prophylaxis, such as ignorance of the indications, fear of bleeding, financial constraints, and lack of effective, rapid, and systematic tools, even though in theory the majority of physicians know when and how to provide prophylaxis.^1^ ^,^ ^28^

Some of the strategies adopted by the institution, such as continuing education, digital tools for stratification of risk and automatic prophylaxis suggestions in the electronic medical prescription software, and creation of a Venous Thromboembolism Prophylaxis Commission (VTPC) have undoubtedly been determinants of the improvement in VTE prophylaxis at the institution. A prospective study by Anderson et al. documented an increase in prophylaxis use from 29 to 52% of patients admitted with potential risk of developing DVT, after implementation of educational strategies with the objective of alerting professionals to the importance of VTE prevalence.^29^

Focusing the work of the VTPC on strategies to raise awareness among attending physicians about the importance of correct stratification, using the tools already available in the electronic prescription system, training attending physicians about use of digital resources, and training the multidisciplinary team to recognize patients at risk (nurses and physiotherapists) could be the way to increase prophylaxis adequacy even further. It is important to encourage replication of studies like this one at other hospital centers, to include larger numbers of patients. Validation of the results presented here could more clearly expose failures in the process of VTE prevention among clinicians and surgeons. It is therefore important to highlight the importance of creating projects that facilitate use of the many different forms of prophylaxis against this disease, combating its elevated morbidity and mortality and reducing the secondary costs.

## CONCLUSIONS

The VTE risk stratification adequacy rate was low, demonstrating the low level of the attending physicians’ awareness of the problem. However, the adequacy rates of prescription of prophylaxis measures were higher than global rates.
