# Supplementary material for: Adequacy of venous thromboembolism risk stratification and prophylaxis in a tertiary university hospital
Source: J Vasc Bras. 2023 Jul 17;22:e20230007. doi: 10.1590/1677-5449.202300071 (PMC10421582; doi:10.1590/1677-5449.202300071)
Supplement: Translation

# Translation: Adequação da estratificação de risco e da profilaxia do tromboembolismo venoso em hospital universitário terciário

**DOI:** 10.1590/1677-5449.202300072

**Authors:** Edvaldo Luiz Ramalli, Marcelo Bellini Dalio, Maurício Serra Ribeiro, Edwaldo Edner Joviliano

**Affiliations:** 1 Universidade de São Paulo – USP, Faculdade de Medicina de Ribeirão Preto, Departamento de Cirurgia e Anatomia, Hospital das Clínicas, Divisão de Cirurgia Vascular e Endovascular, Ribeirão Preto, SP, Brasil.

**Keywords:** tromboembolismo venoso, trombose venosa profunda, embolia pulmonar, fatores de risco, prevenção de doenças

## Abstract

**Contexto:**

O tromboembolismo venoso (TEV) é a terceira doença cardiovascular mais comum e a principal causa de óbito evitável em pacientes internados. Apesar de diretrizes bem estabelecidos na literatura, a profilaxia dessa afecção ainda é subutilizada. Estudos apontam taxa de adequação da profilaxia próxima de 50% mundialmente.

**Objetivos:**

Avaliar a adequação da estratificação de risco e da prescrição de medidas de profilaxia do TEV em um hospital universitário terciário.

**Métodos:**

Estudo transversal observacional realizado através da coleta de dados de prontuário. Foram incluídos pacientes adultos internados em diferentes especialidades e divididos em grupos cirúrgico e clínico. A estratificação de risco de TEV realizada pelos médicos assistentes foi comparada com a realizada pelos médicos pesquisadores, com base nas diretrizes recentes. A prescrição de medidas de profilaxia realizadas pelos médicos assistentes foi comparada com as recomendações das diretrizes, obtendo assim a taxa de adequação da profilaxia.

**Resultados:**

Foram avaliados 400 pacientes, sendo 169 (42,3%) cirúrgicos e 231 (57,7%) clínicos. A taxa geral de adequação da estratificação foi de 50,8%. Nos grupos cirúrgico e clínico, as taxas de adequação foram respectivamente 39,1% e 59,3% (p < 0,0001). A taxa geral de adequação da profilaxia foi de 71,5%, enquanto no grupo cirúrgico foi de 78,1% e no grupo clínico de 66,7% (p = 0,0137).

**Conclusões:**

A adequação da estratificação de risco para TEV foi baixa, demonstrando a pouca conscientização dos médicos assistentes sobre esse problema. No entanto, as taxas de adequação da prescrição de medidas de profilaxia estão superiores às de dados globais.

## INTRODUÇÃO

O tromboembolismo venoso (TEV) engloba a formação de trombos em veias do sistema profundo, ou seja, trombose venosa profunda (TVP), e a migração de êmbolos para a circulação pulmonar, o tromboembolismo pulmonar^1^. Estima-se que na Europa e nos EUA, a incidência anual de TEV seja de 1-3 casos por 1.000 habitantes/ano^2^. Essa afecção é responsável por 300.000 a 600.000 internações por ano^3^, sendo a terceira doença cardiovascular mais comum^2^. Inúmeros são os fatores de risco para o TEV, como: idade, imobilização, TEV prévio, obesidade, varizes, infecção, neoplasia, quimioterapia, insuficiência cardíaca, gravidez e puerpério, uso de anticoncepcional, procedimentos cirúrgicos, entre outros.

A fisiopatologia do TEV ainda não é totalmente conhecida. No entanto, os três pilares propostos por Rudolph Virchow em 1856 (lesão endotelial, estase venosa e hipercoagulabilidade sanguínea) são até hoje aceitos. A lesão endotelial e a estase venosa são facilmente compreensíveis. Os estados de hipercoagulabilidade parecem ser a chave para a compreensão da fisiopatologia. Por meio de mecanismos bioquímicos de sinalização celular ou mesmo regulação da expressão gênica, talvez seja possível entender a razão pela qual algumas pessoas apresentam TEV e outras não, apesar de expostas aos mesmos fatores de risco^2^ ^,^ ^3^.

Devido à grande incidência de TEV em pacientes internados e a seu grande impacto social e financeiro, vários métodos para identificar pacientes com elevado risco foram desenvolvidos. Escores preditivos como de Pádua^4^ e de Caprini et al.^3^ foram adotados para avaliar o risco de TEV em pacientes de internações clínicas e internações cirúrgicas, respectivamente. Com base nesses escores, estratégias para prevenir a ocorrência de TEV têm sido aplicadas^5^ ^,^ ^6^. As profilaxias medicamentosas e mecânica já estão bem estabelecidas em consensos internacionais, tanto em pacientes clínicos como cirúrgicos^2^, baseadas em modelos de estratificação de riscos^3^ ^,^ ^7^ ^,^ ^8^. No entanto, várias publicações nacionais^9^ ^-^ ^19^ e internacionais^20^ ^-^ ^22^ mostram que, no mundo real, cerca de 50% dos pacientes em risco de TEV não estão recebendo profilaxia quando indicada, ou a estão recebendo de forma inapropriada. O estudo transversal ENDORSE avaliou a prevalência de pacientes internados com risco para TEV e a proporção dos que receberam a correta profilaxia. Concluiu-se que aproximadamente metade desses pacientes receberam a profilaxia recomendada pelas diretrizes. Ou seja, mundialmente, a correta profilaxia para TEV é subutilizada^23^.

O objetivo deste estudo é mensurar a situação real da profilaxia do TEV em um hospital terciário universitário brasileiro, avaliando a adequação da estratificação de risco e da prescrição de medidas de profilaxia.

## MÉTODOS

### Desenho e local do estudo

Realizou-se um estudo transversal observacional no Hospital das Clínicas da Faculdade de Medicina de Ribeirão Preto, em São Paulo, Brasil, no período entre 01 de março de 2020 e 30 de junho de 2020. Foram utilizados dados do hospital com aprovação do comitê de ética local, sob o número de processo 15695/2011.

### Participantes

Foram incluídos pacientes adultos com mais de 18 anos, internados nas especialidades de clínica médica, clínica cirúrgica, ginecologia e obstetrícia, ortopedia e traumatologia e terapia intensiva. Foram excluídos pacientes já avaliados uma vez em internação prévia, com obesidade (índice de massa corporal maior que 35 kg/m^2^), gestantes, com contraindicação de anticoagulantes e com indicação de filtro de veia cava.

### Dados coletados

Os dados foram coletados a partir de informações do prontuário eletrônico dos pacientes e respectivas prescrições, sem aviso prévio à equipe médica assistente e sem contato com os pacientes.

### Grupos

Os pacientes foram divididos em dois grupos: clínico e cirúrgico, conforme o tipo de internação. Foram consideradas internações clínicas aquelas em que o paciente não havia sido submetido a nenhum procedimento cirúrgico até o momento da coleta de dados. Internações cirúrgicas foram aquelas em que o paciente tinha sido submetido a algum procedimento em momento prévio à avaliação, independentemente da especialidade médica assistente.

### Estratificação de risco para TEV

Para avaliação do risco e indicação da profilaxia do TEV foi utilizado o escore de risco de Caprini^3^. A estratificação de risco realizada pelos médicos assistentes no momento da admissão hospitalar foi confrontada com uma nova estratificação de risco realizada pelos médicos pesquisadores, com base nos dados presentes no prontuário eletrônico. As estratificações foram classificadas em adequadas ou inadequadas. Foi calculada então a taxa de adequação da estratificação de risco.

### Medidas profiláticas prescritas

A prescrição de medidas de profilaxia do TEV também foi avaliada a partir da prescrição médica diária. Foram consideradas como profilaxia mecânica a prescrição de deambulação precoce, movimentação ativa ou passiva das extremidades inferiores, uso de meias elásticas de compressão graduada e/ou de aparelhos de compressão pneumática intermitente. Da mesma forma, foram consideradas profilaxia medicamentosa a prescrição de heparina não fracionada, heparina de baixo peso molecular ou fondaparinux, conforme as recomendações das diretrizes da Sociedade Brasileira de Angiologia e Cirurgia Vascular (SBACV)^5^ e das diretrizes do American College of Chest Physicians (ACCP)^2^. Os pacientes considerados de baixo risco não precisam, rotineiramente, de profilaxia medicamentosa, mas é recomendada a profilaxia mecânica, quando disponível. Para os de risco moderado, a diretriz sugeriu a utilização de heparina não fracionada (5000 UI de 12/12 horas) ou heparina de baixo peso molecular (enoxaparina 20 mg/dia). Os considerados de alto e altíssimo risco deveriam receber heparina não fracionada (5000 UI de 8/8h) ou heparina de baixo peso molecular (enoxaparina 40 mg/dia). Pacientes clínicos não possuem estratificação intermediária, sendo sempre considerados de baixo ou alto risco, de acordo com recomendações do ACCP.

As medidas de profilaxia prescritas foram comparadas com as recomendações das diretrizes mencionadas acima, tendo como base a estratificação de risco realizada pelos médicos pesquisadores. As medidas profiláticas foram classificadas em adequadas ou inadequadas. Foi calculada então a taxa de adequação da profilaxia do TEV.

### Tamanho do estudo

O tamanho da amostra foi definido em 400 pacientes com base em estudo prévio^21^. Os dados foram coletados de forma aleatória, conforme as internações no período de coleta. A representatividade das especialidades na amostra total respeitou a proporção de leitos de cada especialidade em relação ao total de leitos do hospital.

### Análise estatística

Os dados contínuos foram expressos como média ± desvio padrão e comparados com o teste *t* não pareado. Os dados categóricos foram apresentados como número absoluto (n) e porcentagem (%), comparados com o teste exato de Fischer. Um valor p menor do que 0,05 foi considerado significante.

## RESULTADOS

### Demografia

Foram analisados os prontuários eletrônicos de 400 pacientes, sendo 169 (42,3%) cirúrgicos e 231 (57,7%) clínicos. Os dados demográficos estão demonstrados na Tabela 1. A idade média foi de 56 anos, com ligeira predominância do sexo masculino (56,3%). No grupo cirúrgico, a idade média (60 anos) era maior do que no grupo clínico (53 anos). Não houve diferença significativa entre os grupos em relação ao sexo ou à cor da pele. Entre os pacientes do grupo cirúrgico, predominaram as internações nas especialidades clínica cirúrgica (75%) e ortopedia e traumatologia (16,6%). Nos pacientes do grupo clínico, havia mais internações nas especialidades clínica médica (68,4%), ginecologia e obstetrícia (11, 4,8%) e terapia intensiva (12,1%).

**Tabela 1:** Dados demográficos de todos os pacientes analisados e nos grupos cirúrgico e clínico.

		Geral (n=400)	Cirúrgico (n=169)	Clínico (n=231)	Valor-p
Idade (anos), média ± DP		56 ± 16,04	60 ± 15,51	53 ± 15,84	<0,0001∮
Sexo masculino, n (%)		225 (56,3%)	93 (55%)	132 (57,1%)	0,6844*
Cor da pele, n (%)	Branca	313 (78,3%)	138 (81,7%)	175 (75,8%)	0,1778^*^
	Parda	73 (18,3%)	24 (14,2%)	39 (16,9%)	0,4905^*^
	Preta	13 (3,3%)	7 (4,1%)	16 (6,9%)	0,2815^*^
	Amarela	1 (0,3%)	0 (0%)	1 (0,4%)	1^*^
Especialidade, n (%)	CLM	162 (40,5%)	4 (2,4%)	158 (68,4%)	<0,0001^*^
	CLC	159 (39,8%)	127 (75,1%)	32 (13,9%)	<0,0001^*^
	OT	30 (7,5%)	28 (16,6%)	2 (0,9%)	<0,0001^*^
	GO	12 (3,0%)	1 (0,6%)	11 (4,8%)	0,0165^*^
	UTI	37 (9,3%)	9 (5,3%)	28 (12,1%)	0,0229^*^

DP - desvio padrão; CLM - clínica médica; CLC - clínica cirúrgica; OT - ortopedia e traumatologia; GO - ginecologia e obstetrícia; UTI - unidade de terapia intensiva;

∮- teste *t* não pareado;

* - teste exato de Fisher.

### Estratificação de risco para TEV

A estratificação de risco para TEV realizada pelos médicos assistentes e pelos médicos pesquisadores está descrita na Tabela 2. Os médicos assistentes classificaram a maioria dos pacientes (56,3%) como baixo risco, tanto no grupo cirúrgico (55%) como no grupo clínico (57,1%). Os médicos pesquisadores consideraram a maioria dos pacientes (72%) como alto risco, nos dois grupos: cirúrgico (76,3%) e clínico (68,8%) (p < 0,0001). A taxa geral de adequação da estratificação foi de 50,8%. Nos grupos cirúrgico e clínico, as taxas de adequação foram respectivamente 39,1 e 59,3% (p < 0,0001).

**Tabela 2:** Estratificação de risco para tromboembolismo venoso realizada por médicos assistentes e médicos pesquisadores em todos os pacientes e nos grupos cirúrgico e clínico e taxas de adequação correspondentes.

	Risco para TEV	Médicos assistentes n (%)	Médicos pesquisadores n (%)	Valor-p*	Taxa de adequação (%)
Geral (n=400)	Baixo	225 (56,3%)	89 (22,3%)	<0,0001	50,8%
	Moderado	31 (7,8%)	23 (5,8%)	0,3239	
	Alto	144 (36%)	288 (72%)	<0,0001	
Cirúrgico (n=169)	Baixo	93 (55%)	17 (10,1%)	<0,0001	39,1%
	Moderado	31 (18,3%)	23 (13,6%)	0,2987	
	Alto	45 (26,6%)	129 (76,3%)	<0,0001	
Clínico (n=231)	Baixo	132 (57,1%)	72 (31,2%)	<0,0001	59,3%
	Moderado	-	-	-	
	Alto	99 (42,9%)	159 (68,8%)	<0,0001	

TEV - tromboembolismo venoso;

* - teste exato de Fisher.

### Medidas profiláticas prescritas

A prescrição de medidas profiláticas para TEV pelos médicos assistentes está descrita na Tabela 3. A enoxaparina foi a medida mais utilizada (45%), seguida da heparina não fracionada (19%) e profilaxia mecânica (15%). Em um paciente, foi prescrito rivaroxabana (0,3%), em desacordo com as recomendações das diretrizes seguidas. Todos os pacientes que receberam profilaxia medicamentosa receberam também a profilaxia mecânica. Em 20,5% dos pacientes não foi prescrita nenhuma forma de profilaxia. No grupo cirúrgico, a enoxaparina também foi a mais utilizada (51,5%), seguida da heparina não fracionada (20,1%), profilaxia mecânica (16,6%) e nenhuma profilaxia (11,8%). No grupo clínico, a enoxaparina correspondeu a 40,3%, heparina não fracionada a 18,2%, profilaxia mecânica a 13,9%, fondaparinux a 0,4% e rivaroxabana (*off label*) a 0,4%. Em 26,8% dos pacientes, não foi prescrita nenhuma profilaxia.

**Tabela 3:** Medidas de profilaxia para tromboembolismo venoso prescritas pelos médicos assistentes em todos os pacientes e nos grupos cirúrgico e clínico e taxas de adequação correspondentes.

	Medidas de profilaxia para TEV	Médicos assistentes n (%)	Taxa de adequação (%)
Geral (n=400)	Nenhuma	82 (20,5%)	71,5%
	Mecânica	60 (15%)	
	Enoxaparina	180 (45%)	
	HNF	76 (19%)	
	Rivaroxabana	1 (0,3%)	
	Fondaparinux	1 (0,3%)	
Cirúrgico (n=169)	Nenhuma	20 (11,8%)	78,1%
	Mecânica	28 (16,6%)	
	Enoxaparina	87 (51,5%)	
	HNF	34 (20,1%)	
	Rivaroxabana	0 (0%)	
	Fondaparinux	0 (0%)	
Clínico (n=231)	Nenhuma	62 (26,8%)	66,7%
	Mecânica	32 (13,9%)	
	Enoxaparina	93 (40,3%)	
	HNF	42 (18,2%)	
	Rivaroxabana	1 (0,4%)	
	Fondaparinux	1 (0,4%)	

TEV - tromboembolismo venoso; HNF - heparina não fracionada.

A adequação das medidas profiláticas foi analisada individualmente. A taxa geral de adequação foi de 71,5%, enquanto no grupo cirúrgico foi de 78,1% e no grupo clínico de 66,7% (p = 0,0137). Ao compilar os motivos da não adequação, foram identificados quatro cenários distintos: 1) pacientes que não receberam nenhuma medida de profilaxia; 2) pacientes que possuíam indicação de profilaxia medicamentosa e não receberam; 3) pacientes que receberam profilaxia medicamentosa e não preenchiam critérios para tal; 4) pacientes que receberam medicações ou doses fora das recomendações. Os dados gerais revelam que a omissão da profilaxia foi o erro mais comum, representando 71% das inadequações (81/114), seguido por ausência de profilaxia medicamentosa quando indicado em 28% das inadequações (32/114). Não houve casos nos quais foi prescrito profilaxia medicamentosa quando não havia indicação, e apenas 1% das inadequações foi decorrente de erro de medicação ou dose (1/114).

No grupo cirúrgico, a causa mais comum de erro foram pacientes que não receberam nenhuma profilaxia, correspondendo a 54% das inadequações (20/37). Pacientes que não receberam profilaxia farmacológica quando indicada corresponderam a 46% das inadequações (17/37). No grupo clínico, pacientes que não receberam nenhuma profilaxia corresponderam a 79% das inadequações (61/77), pacientes que não receberam profilaxia farmacológica quando indicada corresponderam a 19% das inadequações (15/77) e pacientes que receberam medicações inadequadas corresponderam a 1% das inadequações (1/77). A Tabela 4 resume esses resultados.

**Tabela 4:** Análise da adequação da estratificação de risco para tromboembolismo venosa e da profilaxia instituída pelos médicos assistentes conforme as recomendações das diretrizes, em todos os pacientes e nos grupos cirúrgico e clínico e taxas de adequação correspondentes.

		Geral (n=400)	Cirúrgico (n=169)	Clínico (n=231)	Valor-*P* *
Taxa de adequação da estratificação de risco para TEV (%)		50,8%	39,1%	59,3%	<0,0001
Taxa de adequação da profilaxia para TEV (%)		71,5%	78,1%	66,7%	0,0137
Não receberam profilaxia adequada, n (%)		114 (28,5%)	37 (21,9%)	77 (33,3%)	0,0137
Motivo da inadequação:					
	Não receberam nenhuma profilaxia, n (%)	81 (71%)	20 (54%)	61 (79%)	0,0080
	Não receberam profilaxia medicamentosa quando indicada, n (%)	32 (28%)	17 (46%)	15 (19%)	0,0068
	Receberam profilaxia medicamentosa inadequadamente, n (%)	0 (0%)	0 (0%)	0 (0%)	1
	Receberam medicações/dose inadequadas, n (%)	1 (1%)	0 (0%)	1 (1%)	1

TEV- tromboembolismo venoso;

* - teste exato de Fisher.

## DISCUSSÃO

A profilaxia adequada do TEV em pacientes internados possui benefícios comprovados, além de ser mais oportuna em relação ao seu tratamento. Idealmente, todos os pacientes em regime hospitalar devem ser estratificados quanto ao risco de desenvolver fenômenos tromboembólicos e receber a profilaxia adequada para preveni-los. O papel dos médicos assistentes nesse processo é fundamental^1^ ^,^ ^24^. O presente estudo demonstrou haver dissonância entre as recomendações de tromboprofilaxia baseadas em evidências científicas e a atual prática clínica. Esse resultado vem ao encontro dos dados da literatura mundial^23^.

No presente estudo, a taxa de adequação da estratificação de risco para TEV realizada pelos médicos assistentes foi de 50,8%. Assim, aproximadamente metade dos pacientes internados não foi estratificada de forma correta, apesar de haver um recurso específico para esse cálculo no sistema de prescrição eletrônica. No grupo cirúrgico, a taxa de adequação foi ainda menor (39,1%). Taxas reduzidas de adequação na estratificação de risco demonstram a baixa adesão dos médicos assistentes ao preenchimento da documentação institucional em relação à prevenção do TEV. Apesar da disponibilidade de um recurso digital que simplifica a estratificação de risco de forma intuitiva e prática, grande parte dos médicos assistentes não preenche de forma adequada as informações no aplicativo e provavelmente apenas pulam os passos, subestimando a estratificação de risco de TEV dos seus pacientes.

Ao comparar as medidas profiláticas do TEV prescritas pelos médicos assistentes com as recomendadas pelas diretrizes da SBACV e do ACCP, com base na estratificação de risco calculado pelos médicos pesquisadores, foi observado que a taxa de adequação foi de 71,5%. Essa taxa é significativamente maior que a relatada em estudos semelhantes, como o ENDORSE, que evidenciou uma taxa global de adequação da profilaxia de 49,8%. Comparando os dados do presente estudo com os dados brasileiros do estudo ENDORSE, observa-se que obtivemos uma taxa de adequação da profilaxia de 78,1% no grupo cirúrgico e de 66,7% no grupo clínico, enquanto que o estudo ENDORSE relata taxas de 46 e 59%, respectivamente. Temos assim um aumento importante na taxa de adequação ao longo do tempo entre esses dados de serviços brasileiros^23^. Em outro estudo brasileiro mais recente, Curtarelli et al. evidenciaram uma taxa de adequação da profilaxia para o TEV de 42,1%, sendo de 37,5% no grupo cirúrgico e de 52,9% no grupo clínico, em outro hospital universitário brasileiro com características semelhantes ao nosso^25^. Um estudo realizado por Bastos et al. demonstrou que a profilaxia medicamentosa para a TVP é empregada em apenas 50% dos pacientes com indicação para utilizá-la, mesmo em hospitais universitários^26^.

A análise dos motivos da não adequação da profilaxia prescrita levaram à compilação dos quatro cenários descritos anteriormente. A primeira situação, na qual o paciente não recebeu nenhuma medida profilática para TEV, foi a mais comum, representando 71% das inadequações, sendo 54% do grupo cirúrgico e 79% do grupo clínico. Pacientes que não receberam profilaxia farmacológica quando indicado representaram 28% dos casos de inadequação, sendo 46% no grupo cirúrgico e 19% no grupo clínico. Pacientes que receberam medicações ou doses inadequadas representaram 1% e em nenhum paciente foi realizada profilaxia medicamentosa sem indicação. Esses dados dos motivos da inadequação da profilaxia prescrita são próximos aos encontrados por Curtarelli et al.^25^ em análise semelhante.

Um dado intrigante levantado no presente estudo é a dissociação da taxa de adequação da estratificação de risco com a taxa de adequação da profilaxia prescrita. Enquanto a taxa de adequação da estratificação foi em torno de 50%, a taxa de adequação da profilaxia foi superior a 70%. No grupo cirúrgico, essa dissociação foi ainda maior, com adequação da estratificação próxima de 40%, enquanto a da profilaxia foi próxima a 80%. Esse achado revela uma baixa adesão dos médicos prescritores ao preenchimento da documentação institucional em relação à prevenção do TEV. Dessa maneira, os métodos auxiliares ao cálculo da estratificação de risco foram subutilizados. A anamnese e o exame clínico adequados servem para dar suporte ao profissional com boa formação médica na tomada de decisão em relação à profilaxia do TEV. Os recursos digitais disponíveis no hospital auxiliam a correta documentação e avaliação do risco individual de cada paciente. Isso pode evitar que a prescrição da profilaxia pelos médicos assistentes seja baseada no empirismo e na percepção pessoal de risco, prevenindo assim a ocorrência de desfechos indesejáveis. Por outro lado, os dados do presente estudo revelam uma maior taxa de adequação da profilaxia prescrita em relação a estudos prévios, demonstrando que a prática clínica de proteção do TEV na instituição tem evoluído favoravelmente.

Considerando a estimativa de que até 75% dos pacientes que apresentam TEV estão internados e que quase metade deles apresenta três ou mais fatores de risco, acredita-se que muitos fatores de risco prevalentes e facilmente identificáveis não costumam ser rastreados rotineiramente^27^. Ao não atentarem para tais fatores de risco como causadores de TEV, os serviços hospitalares deixam de atuar na profilaxia. Tal fato foi ratificado pelos resultados do estudo ENDORSE que concluiu que no Brasil e no mundo a maioria dos pacientes internados está sob risco de desenvolver TEV e que muitos não recebem a profilaxia recomendada^23^. Alguns autores sugerem hipóteses para explicar o manuseio incorreto ou a não utilização de profilaxia, como o desconhecimento das indicações, medo de sangramento, limitações econômicas e ausência de uma ferramenta eficaz e de aplicação rápida e sistemática, apesar de a maioria dos médicos saber, teoricamente, como e quando realizar a profilaxia^1^ ^,^ ^28^.

Algumas estratégias adotadas na instituição, como educação continuada, recursos digitais para estratificação do risco e sugestão de profilaxia automática no software de prescrição médica eletrônica, e a criação de uma Comissão de Profilaxia do Tromboembolismo Venoso (CPTEV) certamente foram determinantes nessa melhora da profilaxia do TEV na instituição. Um estudo prospectivo de Anderson et al. documentou aumento no uso da profilaxia de 29 para 52% em pacientes internados com risco potencial para desenvolver TVP, após a instituição de estratégias educacionais com o propósito de alertar os profissionais para a importância da prevalência do TEV^29^.

Focar o trabalho da CPTEV em estratégias de conscientização dos médicos assistentes sobre a importância da estratificação adequada, utilizando ferramentas já disponíveis no sistema de prescrição eletrônica, capacitação dos médicos assistentes sobre a utilização dos recursos digitais, capacitação da equipe multidisciplinar no reconhecimento de pacientes em risco (enfermeiros e fisioterapeutas) podem ser o caminho para aumentar ainda mais a adequação da profilaxia. É importante que se estimule a replicação de estudos como este em outros centros hospitalares, para incluir um número maior de pacientes. A validação dos resultados aqui apresentados pode expor com mais clareza as falhas do processo de prevenção do TEV entre clínicos e cirurgiões. Dessa forma, busca-se destacar a importância da criação de projetos que facilitem o uso das diversas formas de profilaxia para essa doença, visando a combater sua alta morbimortalidade e diminuir seus custos secundários.

## CONCLUSÃO

A adequação da estratificação de risco para TEV foi baixa, demonstrando a pouca conscientização dos médicos assistentes sobre o problema. No entanto, as taxas de adequação da prescrição de medidas de profilaxia estão superiores às de dados globais.
